# SARS-CoV-2 viral dynamics in a placebo-controlled phase 2 study of patients infected with the SARS-CoV-2 Omicron variant and treated with pomotrelvir

**DOI:** 10.1128/spectrum.02980-23

**Published:** 2024-01-10

**Authors:** Katyna Borroto-Esoda, David Wilfret, Xiao Tong, Andrew Plummer, Brian Kearney, Ann D. Kwong

**Affiliations:** 1 Pardes BioSciences Inc., Carlsbad, California, USA; Emory University School of Medicine, Atlanta, Georgia, USA

**Keywords:** SARS-CoV-2, Omicron, viral dynamics, rapid antigen test, infectious virus assay, qRT-PCR

## Abstract

Current guidelines recommend that individuals with moderate COVID-19 disease isolate for 5 days after the first appearance of symptoms or a positive SARS-CoV-2 test. It would be useful to understand the time course of infectious virus production and its correlation with virus detection using a rapid antigen test (RAT) or quantitative reverse transcriptase (qRT)-PCR. In a phase 2 study, 242 vaccinated patients with COVID-19 and at low risk for progression to severe disease initiated 5 days of treatment with pomotrelvir (PBI-0451, a SARS-CoV-2 main protease inhibitor) or placebo within 5 days after symptom onset. The primary endpoint, the proportion of subjects with SARS-CoV-2 viral titers below the limit of detection on Day 3 of treatment in the pomotrelvir versus placebo groups, was not met. No between-group differences in SARS-CoV-2 clearance or symptom resolution or alleviation were observed. Additional analyses evaluated the dynamics of SARS-CoV-2 replication in mid-turbinate nasal swabs and saliva samples using infectious virus assay (IVA), RAT, and qRT-PCR. SARS-CoV-2 cleared rapidly, with negative results first determined by IVA (TCID_50_ below the limit of detection), followed by the RAT (negative for SARS-CoV-2 N antigen), and qRT-PCR (RNA below the limit of detection), which suggests that delayed initiation of treatment (up to 5 days after symptom onset) may have contributed to the lack of treatment response. Symptom resolution lagged behind viral clearance assessed by IVA and RAT. These data support reliance on a negative RAT to determine when an individual is no longer producing infectious virus and may end isolation.

**IMPORTANCE:**

A phase 2 double-blind, placebo-controlled study was performed evaluating pomotrelvir, a SARS-CoV-2 Mpro inhibitor, compared with placebo in 242 non-hospitalized, vaccinated, symptomatic adults with COVID-19 (Omicron). No improvement in the decrease of viral replication or relief of symptoms was observed between the two groups when treatment was initiated ≥3 days after symptom onset. These results suggest that future COVID-19 antiviral studies using a similar patient population may need to initiate treatment earlier, like influenza studies. This is the first study to prospectively evaluate SARS-CoV-2 viral dynamics and the time to viral clearance in a significant number of patients using concurrently obtained results from an infectious virus assay, a rapid antigen test (RAT), and a qRT-PCR assay over a 15-day time course. These results suggest that a negative RAT assay is a good indicator of loss of infectious virus and the ability to return to normal activities.

## INTRODUCTION

Infection with SARS-CoV-2 has resulted in widespread economic and social disruption as governments struggle to decrease the spread of the highly infectious virus that can produce asymptomatic infection, as well as severe disease, hospitalization, and death. The B.1.1.529 (Omicron) variant of SARS-CoV-2 was first identified in the United States on 1 December 2021 and became the predominant circulating strain, representing >95% of samples identified in the Centers for Disease Control and Prevention’s (CDC) national genomic surveillance program by mid-January 2022 (1). Factors contributing to the rapid emergence and continued prevalence of Omicron include a higher rate of transmissibility and infectivity in humans compared with earlier SARS-CoV-2 lineages (2, 3), resistance to neutralization by monoclonal antibodies, and, to a large extent, vaccine-elicited antibodies (4). This has led to high rates of breakthrough infections among both vaccinated and previously infected individuals (5).

Although individuals infected with SARS-CoV-2 Omicron variants tend to experience milder symptoms and are at lower risk for severe outcomes and hospitalization compared with earlier variants (6, 7), confirmed SARS-CoV-2 infections continue to result in thousands of hospitalizations per day and account for 1.5% of deaths in the United States (https://covid.cdc.gov/covid-data-tracker/#datatracker-home). The majority of hospitalizations and deaths are in patients with risk factors that include but are not limited to, hypertension, obesity, immune suppression, and chronic lung disease (https://www.cdc.gov/coronavirus/2019-ncov/hcp/clinical-care/underlyingconditions.html#print) (8).

Current CDC guidelines recommend initiation of treatment within 3–5 days of development of COVID-19 symptoms (https://www.cdc.gov/coronavirus/2019-ncov/your-health/treatments-for-%20severe-illness.html). Direct-acting antiviral small molecule protease/polymerase inhibitors (i.e., nirmatrelvir/ritonavir, molnupiravir, and remdesivir) have received regulatory emergency use authorization or full approval for the treatment of COVID-19 in the United States and other regions [Paxlovid (Pfizer) 2023, Lagevrio (Merk) 2021, and Velkury (Gilead Sciences) 2021] but are indicated only for patients who are at high risk for progression to severe COVID-19, including hospitalization or death; they are not authorized for treatment of standard-risk, otherwise healthy patients.

Given the rapid clearance of Omicron, a 3- to 5-day delay in the initiation of treatment in an otherwise healthy patient population with a low risk of severe disease progression may not result in noticeable improvements in viral clearance or symptom improvement. Herein, we present the results of a phase 2 double-blind, placebo-controlled study evaluating the virologic effect, safety, and efficacy of pomotrelvir, an orally bioavailable direct-acting antiviral inhibitor of the main protease (M^pro^) of SARS-CoV-2 (9), compared with placebo in non-hospitalized, symptomatic adults with COVID-19 who were not at high risk of progressing to severe disease. While no statistically significant difference in the clearance of SARS-CoV-2 was observed between the treatment arms, the use of three independent virologic assays allowed us to closely monitor the time to viral clearance in this patient population.

SARS-CoV-2 detection by RT-PCR is generally considered the gold standard for the diagnosis of COVID-19 (10), and the performance of the commercially available SARS-CoV-2 rapid antigen tests (RATs) has been evaluated by comparing RAT and RT-PCR results from concordant nasal swab samples (11). The RT-PCR and RAT tests are commonly used to track the course of infection to determine when it is appropriate to end isolation. However, neither of these assays determines the presence of infectious viruses. Previous studies evaluating the performance of RAT and RT-PCR assays compared to viral culture [i.e., infectious virus assay, IVA (12, 13)] were conducted prior to the onset of Omicron lineage. Chu and colleagues (12) executed a study from January to May 2021, in which the SARS-CoV-2 lineages detected were 56% Alpha, 16% Epsilon, and 4% Gamma. Only 23 of the 225 patients in the study were included in a comparison of antigen, NP swab, and viral culture samples, which were taken for 7 days following enrollment. However, the authors do not describe whether these samples were taken concurrently. In their study, the antigen test sensitivity was 64% and 84% when compared with same-day RT-PCR and viral culture results, respectively. Kirby and colleagues (13) compared the antigen, RT-PCR, and viral culture results from 189 samples collected from March to June 2021 (no Pango lineage was described), which were selected based solely on the viral load distribution by RT-PCR. In their study, when the viral load was >10^6^ copies/mL, a positive antigen test was >95% positive in the viral culture assay for the same sample. The study presented in this communication is the first clinical study of COVID-19 patients to prospectively test and compare concurrently obtained IVA, RAT, and RT-PCR assay results from 242 patients, which describes the viral dynamics of a SARS-CoV-2 infection over a full 15-day time course.

## RESULTS

### Patient disposition

Two hundred eighty-nine patients were screened at 33 sites in the United States from 21 September through 27 December 2022, and 242 eligible patients were randomized to the intent-to-treat (ITT) population (162 patients to the pomotrelvir group and 80 patients to the placebo group). Overall, 231 patients (95.5%) completed 5 days of twice-daily dosing and 10 patients (4.1%) prematurely discontinued the study drug. In the pomotrelvir group, eight patients (4.9%) prematurely discontinued the study drug. Five patients (3.1%) were study drug noncompliant, one subject (0.6%) withdrew from the study prior to completing study drug dosing, one patient (0.6%) discontinued the study drug due to an adverse event (nonserious hemorrhoidal hemorrhage), and one patient (0.6%) discontinued the study drug due to pregnancy. In the placebo group, two patients (2.5%) prematurely discontinued the study drug (one subject withdrew consent and one subject at the investigator’s discretion). A total of 227 patients (*n* = 149 pomotrelvir and *n* = 77 placebo) completed the study through Day 28 (Fig. S1).

### Demographics

Clinical characteristics were similar between the two groups (Table S1). The median age was 43 years and 47.5% were male. Most patients were white (84.7%), of Hispanic ethnicity (79.8%), and randomized within 3 days of symptom onset (81.8%). The majority (69%) had evidence of previous SARS-CoV-2 infection as determined by the detection of SARS-CoV-2 antibodies to the N antigen at baseline. At baseline, the incidence of 14 targeted COVID-19 symptoms was similar for patients in the pomotrelvir and placebo groups. The baseline viral load values obtained from mid-turbinate (MT) nasal swab or saliva samples were similar between the two groups: 5.34 and 5.13 log_10_ copies/mL for MT nasal swab samples and 4.71 and 4.93 log_10_ copies/mL for saliva samples. At baseline, the majority (64.9%) of patients were negative by IVA; therefore, only 85 patients (35.1%) who had a positive SARS-CoV-2 result based on IVA were included in the modified intent-to-treat virology (mITTV) population: (32.7%) in the pomotrelvir group and 40% in the placebo group. Viral titers were also similar between the two groups: 2.01 and 2.13 log_10_ TCID_50_/mL for the pomotrelvir and placebo groups, respectively (Table S1). Whole-genome sequencing data were obtained from 123 patients at baseline (84 patients in the pomotrelvir group and 39 patients in the placebo group). All sequenced patients were infected with the Omicron variant of SARS-CoV-2. The predominant lineage observed was BQ.1/BQ.1.1 (52.4% in the pomotrelvir group and 61.5% in the placebo group) and sub-lineages thereof (Table 1).

**TABLE 1:** SARS-CoV-2 analysis

SARS-CoV-2 lineage	Pomotrelvir (*n* = 84) *n* (%)	Placebo (*n* = 39) *n* (%)
BQ.1/BQ.1.1	44 (52.4)	24 (61.5)
BA.5	14 (16.7)	4 (10.3)
XBB	12 (14.3)	5 (12.8)
BF	5 (6.0)	4 (10.3)
BA.4.6	3 (3.6)	0
EF.2	2 (2.4)	0
BE.9	1 (1.2)	1 (2.6)
BN.1.5	1 (1.2)	1 (2.6)
BW.1.1.1	1 (1.2)	0
CV.1	1 (1.2)	0

### SARS-CoV-2 viral titer and viral load

**Fig 1:**
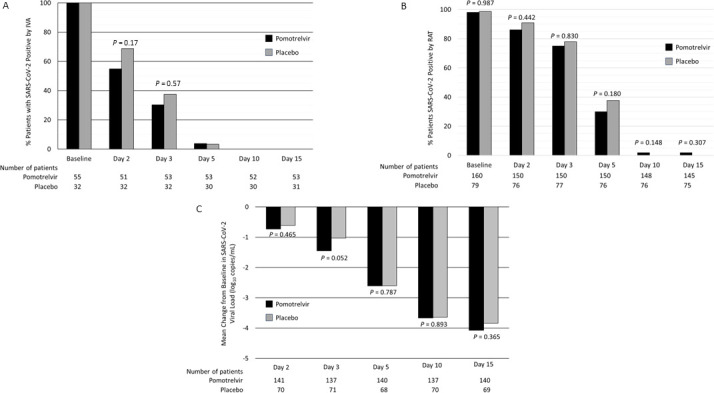
Proportion of patients positive for SARS-CoV-2 by time point assessed by IVA (A) and RAT (B) and change from baseline in SARS-CoV-2 viral load (qRT-PCR) by time point (C). (A) Proportion of patients with positive SARS-CoV-2 by IVA (mITTV analysis set), (**B**) proportion of patients with positive SARS-CoV-2 by RAT (mITT analysis set), and (**C**) mean change from baseline in SARS-CoV-2 viral load (quantitative reverse transcriptase-PCR, ITT analysis set).

The proportion of subjects in the mITTV analysis set with undetected SARS-CoV-2 infectious virus on Day 3 (i.e., primary endpoint) was not significantly different between the pomotrelvir and placebo treatment groups (risk difference 6.2, 95% CI −14.12, 26.61, *P* = 0.5694). By Day 5 of treatment, 96.2% of patients in the pomotrelvir group and 96.7% of patients in the placebo group had SARS-CoV-2 viral titers below the limit of detection, and viral titers remained below the limit of detection at all subsequent time points (Fig. 1A). Patients included in the modified intention-to-treat (mITT) analysis set had a positive SARS-CoV-2 RAT at baseline. On Day 5 of treatment, the proportion of patients with a negative RAT by MT nasal swab was slightly higher in the pomotrelvir group (70.7%) compared with the placebo group (61.8%). However, the difference between the pomotrelvir and placebo groups was not significantly different (risk difference 9.3, 95% CI −3.64, 22.23, *P* = 0.1552). By Day 28, all patients were negative for SARS-CoV-2 by RAT (Fig. 1B).

For the mITT analysis set, baseline SARS-CoV-2 viral load by quantitative reverse transcriptase (qRT)-PCR from MT nasal swabs was similar for patients in the pomotrelvir and placebo groups (5.34 ± 2.148 and 5.13 ± 2.465 log_10_ copies/mL, respectively). Viral RNA levels decreased at a similar rate for both groups, with decreases from baseline −2.61 ± 1.615 and −2.60 ± 2.060 log_10_ copies/mL in the pomotrelvir and placebo groups, respectively, on Day 5 (*P* = 0.7873). By Day 15, 97.2% of patients in the pomotrelvir group and 96.0% of patients in the placebo group were below the limit of detection for SARS-CoV-2 by qRT-PCR (Fig. 1C).

Baseline and post-baseline saliva viral load (qRT-PCR) results were obtained from 95 patients in the pomotrelvir group and 48 patients in the placebo group (data not shown). Saliva viral load at baseline was similar for the pomotrelvir (4.71 ± 1.643 log_10_ copies/mL) and placebo (4.93 ± 1.401 log_10_ copies/mL) groups. On Day 5, there was a significant difference in the change from baseline saliva viral load between the pomotrelvir and placebo groups [−2.73 ± 1.658 log_10_ copies/mL versus −1.83 ± 1.651 log_10_ copies/mL for pomotrelvir and placebo, respectively (*P* = 0.0057)]. No significant differences in change from baseline saliva viral load were observed between the groups at any other time point. By Day 15, 95.8% of patients in both groups were below the limit of detection for SARS-CoV-2 in saliva samples.

### Virologic rebound

**Fig 2:**
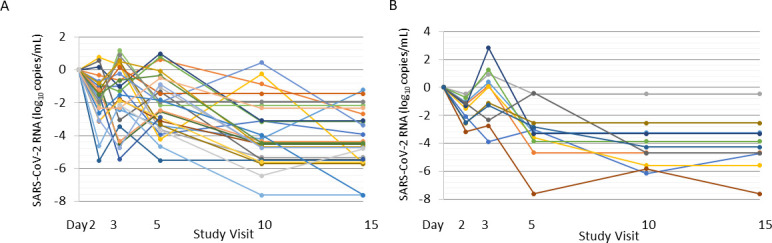
Change from baseline in SARS-CoV-2 viral load over time for patients with virologic rebound in the pomotrelvir (A) and placebo (B) groups. Individual graphs representing a change from baseline in SARS-CoV-2 RNA by treatment group for patients with virologic reboundt.

The proportion of patients with virologic rebound detected by qRT-PCR from MT nasal swab samples was evaluated as part of an exploratory endpoint. Virologic rebound was defined as (i) SARS-CoV-2 RNA greater than the lower limit of detection after being less than the limit of detection or (ii) at least 1 log_10_ increase from nadir in SARS-CoV-2 RNA copy number. Among patients with detectable SARS-CoV-2 by qRT-PCR at baseline and at least two subsequent time points, 21.1% (38/180) experienced virologic rebound, with similar incidence in the pomotrelvir (21.6%; 27/125) and placebo (20.0%; 11/55) groups; the between-group difference was not statistically significant (*P* = 0.86). For the majority of these patients (81.6%; 31/38), virologic rebound occurred on or before Day 5. For 6/38 (15.8%) patients who experienced virologic rebound (three patients each in the pomotrelvir and placebo groups), concurrent saliva samples also met the criteria for rebound. Virologic rebound by qRT-PCR was not associated with a contemporaneous rebound by RAT or with a rebound of clinical symptoms. All virologic rebounds were transient in that the viral load decreased and became undetectable at subsequent time points (Fig. 2).

### COVID-19 symptom alleviation and resolution

Across treatment groups, the median time to sustained alleviation (8 days) and sustained resolution (10–11 days) of all 14 targeted COVID-19 symptoms and subsets of symptoms was similar, and no statistically meaningful between-group differences were observed (Table S2). No patients reported a COVID-19-related medical visit or hospitalization. The proportion of patients with symptom rebound was greater in the pomotrelvir group (9.2%) compared with the placebo group (2.6%) for symptoms that lasted ≥1 day. However, for symptoms lasting ≥2 consecutive days, the proportion of patients with symptom rebound was similar for the pomotrelvir (2.6%) and placebo (1.3%) groups. No patients reported a severe symptom or required hospitalization at the time of symptom rebound.

### Safety

Pomotrelvir was safe and well tolerated by patients; 13.7% (22/161) in the pomotrelvir group and 5.0% (4/80) in the placebo group had at least one treatment-emergent AEs (TEAE), most of which were Grade 1 (mild) in severity. The most common TEAEs in the pomotrelvir group were gastrointestinal disorders (9.3%, 15/161) and included nausea (4.3%, 7/161), diarrhea (1.9%, 3/161), and abdominal pain and food poisoning (1.2%, 2/161 each). Laboratory parameters remained consistent throughout the study with similar changes from baseline between the pomotrelvir and placebo groups. A total of 9.9% (16/161) of patients in the pomotrelvir group and 8.9% (7/80) in the placebo group had at least one Grade 3 or 4 treatment-emergent chemistry laboratory abnormality, none of which were considered clinically significant. No individual vital sign measurement or electrocardiogram finding was considered clinically significant.

### Summary of clinical results

Overall, the results of the virologic and clinical analyses demonstrated that treatment with pomotrelvir tablets 2× 750 mg/day for 5 days did not result in significant activity against SARS-CoV-2 compared with placebo in patients who were vaccinated (of whom 69% were previously infected), symptomatic adults who were not at risk of progression to severe COVID-19.

### Pooling of data for comparisons of SARS-CoV-2 detection and sampling methods

Because no significant differences were observed between the pomotrelvir and placebo treatment groups in the virologic analyses, the IVA, RAT, and qRT-PCR results obtained from patients in the mITT analysis set were pooled to evaluate any correlation between the three assays during the natural course of SARS-CoV-2 Omicron infection in this population of otherwise healthy, vaccinated patients, 69% (167/242) of whom had evidence of previous COVID-19 infection. In particular, a comparison of the IVA titer to RAT results in samples from MT nasal swabs and to qRT-PCR results from MT nasal swabs and saliva samples were evaluated to assess their utility in determining when a patient is no longer contagious and can return to normal activities.

### Viral dynamics of SARS-CoV-2 clearance

**Fig 3:**
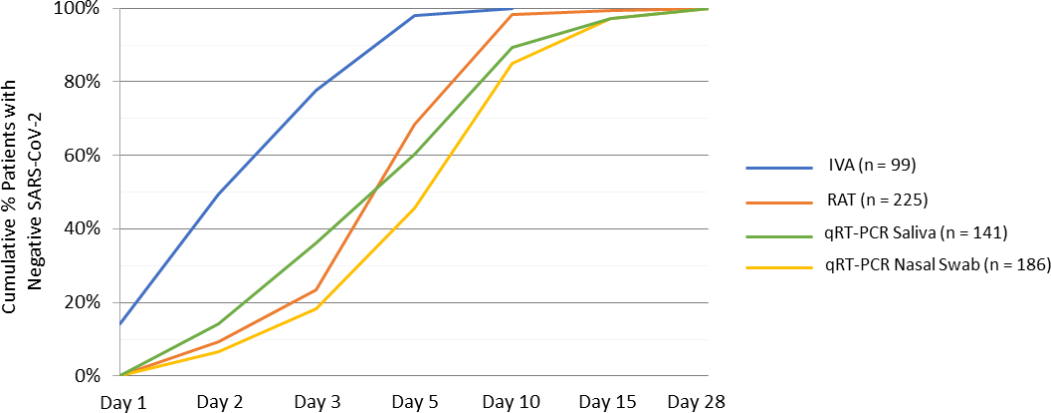
Cumulative proportion of patients with negative SARS-CoV-2 result by IVA, RAT, and qRT-PCR at each time point evaluated. All patients in the mITT analysis set were included in this analysis. Numbers in parenthesis indicate the number of patients with a positive test by IVA, RAT, and qRT-PCR (saliva and nasal swabs).

The time points at which patients had their first negative SARS-CoV-2 result by IVA, RAT, and qRT-PCR were calculated based on the results from MT nasal swabs and saliva samples. The first negative results were detected by IVA on Day 1, with 14.1% (14/99) of evaluated patients negative for SARS-CoV-2 compared with no negative patients detected by RAT or qRT-PCR (Table 2). For the greatest proportions of evaluated patients, their first negative SARS-CoV-2 result occurred on Day 2 by IVA (35.4%) followed by Day 5 by RAT (44.9%) and Day 10 by qRT-PCR of nasal swab (39.2%) and saliva (29.1%). The cumulative proportion of patients with negative SARS-CoV-2 by IVA, RAT, and qRT-PCR at each time point evaluated is shown in Fig. 3.

**TABLE 2:** First time point with negative SARS-CoV-2 result by IVA, RAT, and qRT-PCR

% Patients first negative by	Study day						
	1	2	3	5	10	15	28
IVA (*n* = 99)	14.1	35.4	29.3	20.2	2	0	0
RAT (*n* = 225)	0	9.3	14.2	44.9	29.8	1.3	0.4
qRT-PCR by nasal swab (*n* = 186)	0	6.5	11.8	27.4	39.2	12.4	2.7
qRT-PCR by saliva (*n* = 141)	0	14.2	22.2	24.1	29.1	7.8	2.8

### Correlation between saliva and MT nasal swab viral load

**Fig 4:**
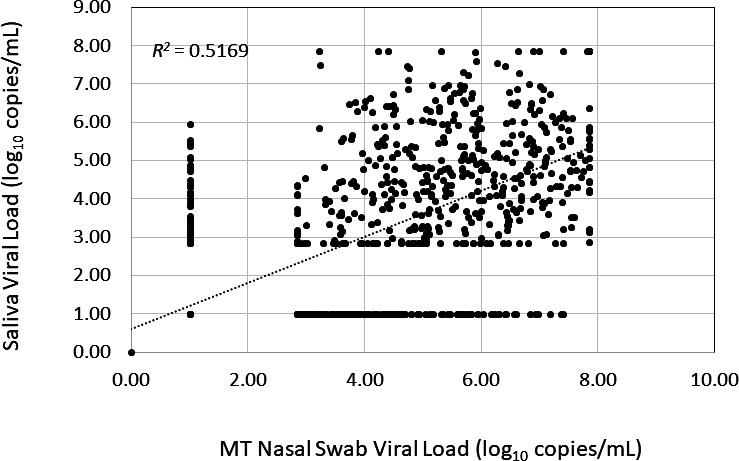
Correlation between SARS-CoV-2 viral load detected in paired saliva and MT nasal swab samples assessed by qRT-PCR. Correlation based on pooled qRT-PCR results obtained for concurrent saliva and nasal samples collected from baseline to Day 28 (*n* = 1,432).

**Fig 5:**
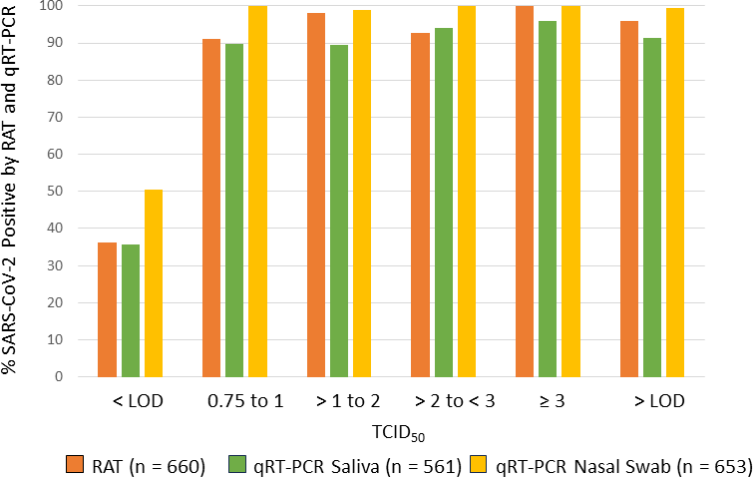
Proportion of SARS-CoV-2 positive samples assessed by RAT and qRT-PCR by TCID_50_ of matching SARS-CoV-2 positive samples assessed by IVA. Data obtained from concordant samples through Day 15 for patients with a positive SARS-CoV-2 IVA result obtained within 72 hours of screening.

We pooled the viral load results from 1,432 paired nasal swabs and saliva samples collected from baseline to Day 28 to evaluate the concordance in viral load from the two sampling methods. Linear regression analysis revealed a moderate correlation in viral load from nasal swabs and saliva samples (*r*^2^ = 0.5169, Fig. 4). At each time point evaluated, the absolute viral load values were significantly different between the saliva and nasal swab samples (*P* values < 0.0001). An analysis that included only those samples with results within the linear range of each assay (*n* = 415) also demonstrated a significant difference in viral loads (*P* < 0.0001). At baseline, saliva viral loads (4.981 ± 1.486 log_10_ copies/mL) were lower than nasal swab viral loads (6.255 ± 1.444 log_10_ copies/mL) (*P* < 0.001); changes from baseline in saliva and nasal swab viral loads were significantly different at multiple time points. In addition to the significant scatter, the lack of concordance was most dramatic at the lowest range of sensitivity for each assay limit of detection (LOD) where the saliva counterpart assay detected significant viral loads ranging from <3 to 6 log_10_ copies, and the swab counterpart assay samples detected viral loads ranging from <3 to >7 log_10_ copies/mL. Not all patients in the study initiated treatment (i.e., Day 1) on the same day as the screening visit; therefore, IVA, RAT, and qRT-PCR results were compared for 105 patients who tested positive for SARS-CoV-2 by IVA within 72 hours after screening. The total number of samples with paired results through Day 15 were 660 RAT/IVA, 561 saliva qRT-PCR/IVA, and 653 nasal swab qRT- PCR/IVA. Overall, 96% of nasal swab samples that were positive for SARS-CoV-2 by IVA was also positive by RAT, and 100% of IVA samples with TCID_50_ greater than 3 log10 was positive by RAT; 99.5% of nasal swab samples and 91.0% of saliva samples that were positive by IVA were also positive by qRT-PCR. Among samples that were below the limit of detection for SARS-CoV-2 by IVA, 36% was positive by RAT and saliva qRT-PCR; 50% of samples that were below the limit of detection for SARS-CoV-2 by IVA was positive by MT nasal swab qRT-PCR (Fig. 5).

**Fig 6:**
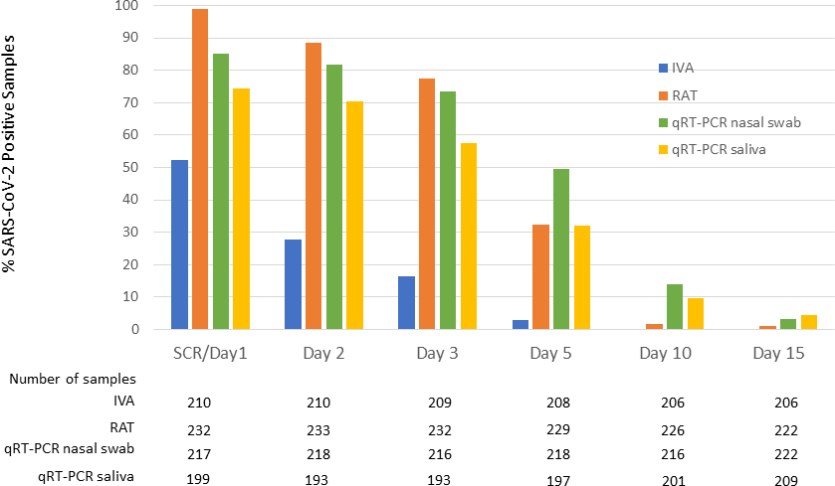
Proportion of SARS-CoV-2 positive samples by assay and time point. Data from all subjects randomized in the study (ITT analysis set, *n* = 242).

**Fig 7:**
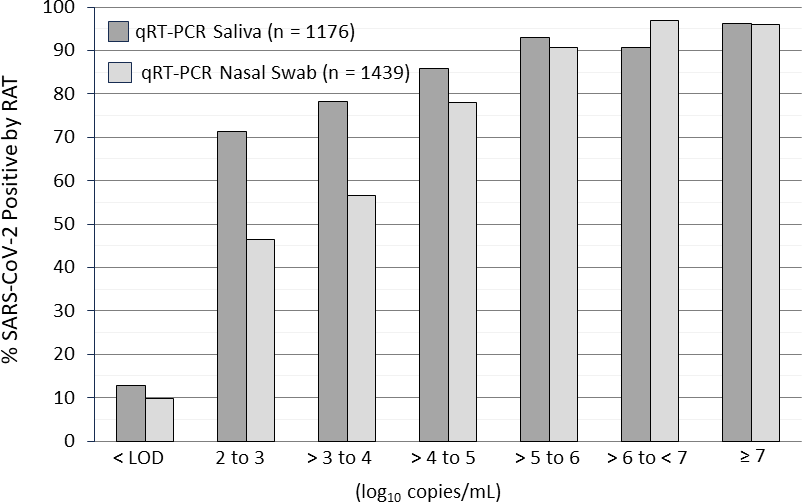
Proportion of SARS-CoV-2 positive RAT samples by viral load (log_10_ copies/mL) of matching saliva and nasal sample. Data from 1,439 nasal samples and 1,176 saliva samples with positive qRT-PCR results.

Throughout the study, the proportion of samples that were positive for SARS-CoV-2 was consistently higher by RAT and qRT-PCR compared with IVA. On Day 5, 3% of nasal swab samples was positive by IVA compared with 32% and 50% by RAT and qRT-PCR, respectively, and 32% of saliva samples by qRT-PCR. On Day 10, 0%, 2%, and 15% of nasal swab samples were positive by IVA, RAT, and qRT-PCR, respectively, and 10% of saliva samples was positive by qRT-PCR (Fig. 6). We compared RAT and qRT-PCR results for patients with contemporaneously collected MT nasal swabs and saliva samples through Day 15. Results from 1,439 nasal swab samples and 1,176 saliva samples were included in the analysis. The proportion of samples positive for SARS-CoV-2 by RAT increased with increasing viral load by qRT-PCR; 96% of nasal swabs and saliva samples that yielded viral loads greater than seven log_10_ copies/mL by qRT-PCR were positive by RAT (Fig. 7). The correlation between RAT and qRT-PCR was slightly greater for nasal swab samples (*r*^2^ = 0.9826) compared with saliva samples (*r*^2^ = 0.8644).

### Correlation of RAT and qRT-PCR for early SARS-CoV-2 detection

The concordance of SARS-CoV-2 detection by RAT and qRT-PCR was evaluated for 216 patients with a positive RAT test at baseline for whom baseline nasal swabs and saliva qRT-PCR data were available. The concordance between positive RAT results and positive qRT-PCR results in contemporaneous baseline samples was 89.4% and 85.6% for nasal swabs and saliva samples, respectively. When the qRT-PCR results from baseline, Day 2, and Day 3 were combined for 203 subjects with available data, the concordance was 92.1% and 88.1% for nasal swabs and saliva samples, respectively (Table 3).

**TABLE 3:** Concordance between SARS-CoV-2 positive RAT at baseline and positive MT nasal swabs and saliva samples by qRT-PCR through Day 3

SARS-CoV-2 positive by qRT-PCR from nasal swabs	No. of patients	Baseline	Baseline/Day 2/Day 3
Positive RAT at baseline	225		
Missing baseline nasal swab qRT-PCR	9		
RAT positive with nasal swab qRT-PCR	216	193	199
% Concordance between RAT and nasal swab qRT-PCR		89.4	92.1
**SARS-CoV-2 positive by qRT-PCR from saliva sample**			
Positive RAT at baseline	225		
Missing baseline saliva qRT-PCR	22		
RAT positive with saliva qRT-PCR	203	163	178
% Concordance between RAT and saliva		85.6	88.1

## DISCUSSION

Pomotrelvir is an orally bioavailable direct-acting 3C-like protease inhibitor of SARS-CoV-2 main protease. This phase 2 study evaluated the antiviral activity, clinical efficacy, and safety of 5 days of pomotrelvir 700 mg twice daily in vaccinated, non-hospitalized, symptomatic adults with COVID-19, who were at standard risk of progressing to severe disease, 69% of whom had evidence of previous infection with SARS-CoV-2. The prespecified primary efficacy endpoint for the study was based on the proportion of subjects below the limit of detection for infectious SARS-CoV-2 on Day 3 of treatment by IVA from MT nasal swabs. The pre-specified statistical analysis plan assumed a 40% positivity rate for SARS-CoV-2 by IVA at the start of treatment (i.e., baseline) to detect a difference between the groups. However, in our study, only 35% of the randomized patients had a positive SARS-CoV-2 result based on IVA. This result is lower than that reported with other SARS-CoV-2 variants. For example, in the phase 2 study of molnupiravir, which enrolled patients prior to the appearance of the first variant of concern, 43.5% of the patients was positive at baseline by IVA (14), whereas in the phase 2b study of ensitrelvir where the majority of patients were infected with the Omicron BA.1 variant, 80% were positive at baseline by IVA (15). The impact of the relatively low incidence of positive IVA at baseline on treatment response is unknown. However, we did not observe any statistically significant differences in other virologic endpoints (RAT and/or PCR) where greater than 90% of the patients had a positive result at baseline, nor were there any significant differences observed in the clinical endpoints between the pomotrelvir and placebo group in this study. We observed multiple instances of transient virologic rebound in approximately 20% of patients by MT nasal swab qRT-PCR. The incidence of rebound was similar for both treatment groups; and unlike results reported for Paxlovid-treated, high-risk patients (16), rebound was more frequent on or before Day 5. Patients in both the pomotrelvir and placebo groups reported a higher incidence of COVID-19 symptoms at baseline compared with other studies (17). The rapid decline in viral titers and viral load and the short time to alleviation and resolution of symptoms was consistent with a rapid and robust immune response to infection.

This is the first phase 2 clinical study of COVID-19 patients to utilize IVA, RAT, and qRT-PCR assays concurrently to prospectively evaluate SARS-CoV-2 viral dynamics and compare results across assays. For the initial diagnosis of COVID-19, SARS-CoV-2 detection by PCR is generally considered the gold standard (10). The performance of the commercially available SARS-CoV-2 RAT assays has been evaluated by comparing RAT results with RT-PCR results from nasal swab samples (11). In this study, the nearly 100% concordance observed between RAT and IVA assays and the low incidence of positive RAT results following a negative IVA test suggest that RATs could be used as a surrogate for IVA in determining SARS-CoV-2 infectivity. By contrast, the more sensitive qRT-PCR assay detected SARS-CoV-2 in MT nasal swabs for up to 15 days after the patients were negative by IVA (and presumably no longer infectious). Our results are in agreement with those observed in previous studies evaluating the performance of RAT and RT-PCR assays compared to viral culture, which also demonstrated a good correlation between the RAT and IVA in patients infected with earlier lineages of SARS-CoV-2 (12, 13). However, unlike our current study, these studies were limited in the number of patients with concordant samples as well as the number of time points tested over the course of infection, thereby limiting the interpretation of the use of the RAT for the determination of self-isolation.

Given the discomfort of MT nasal swab sampling, an exploratory objective of the study was to use saliva samples to evaluate SARS-CoV-2 assessed by qRT-PCR over time. In general, we did not observe a good correlation between the saliva and nasal swab qRT-PCR results in these Omicron-infected patients. At baseline, the absolute viral load from saliva samples was significantly lower than that from MT nasal swab samples and was significantly different from nasal swab samples at each time point evaluated. These differences in sampling method were apparent in the evaluation of virologic rebound, whereby fewer than 20% of patients with rebound determined by MT nasal swab also had a rebound determined by saliva. Differences between the two sampling methods may also have contributed to our observation of a significant decrease in saliva viral load on Day 5 between the pomotrelvir and placebo-treated group; no significant differences in treatment response were observed in all other virologic or clinical endpoints. Overall, our results suggest that the MT nasal swab sampling is the more sensitive assay for the determination of SARS-CoV-2 infection. These findings are in agreement with those observed in a meta-analysis conducted by Lee et al. evaluating the performance of SAR-CoV-2 detection by PCR using alternative sample types (e.g., saliva and oral pharyngeal), which demonstrated lower positivity rates for saliva as compared to nasal swabs (18).

The rigorous virologic assessments demonstrated a rapid resolution of SARS-CoV-2 Omicron infection in the study population of vaccinated, low-risk individuals, the majority of whom had evidence of previous SARS-CoV-2 infection. For the majority of patients, clearance of infectious virus occurred within 3 days of randomization into the study, and clearance of viral RNA by qRT-PCR occurred within 10 days. These results are consistent with animal models of SARS-CoV-2 infection wherein the infectious period was short and correlated with the detection of infectious virus but not viral RNA (19). Since symptoms are the sequelae of the innate immune response to infection and all patients had to present with symptoms to enroll in the study and no patients developed long COVID, it makes sense that the resolution of COVID-19 symptoms observed in this study occurred after the IVA and RAT assays became negative; the median time to sustained alleviation was 8 days and sustained resolution was 10–11 days after randomization.

When this study was initiated in September 2022, the window for initiation of treatment among high-risk COVID-19 patients with approved antiviral agents was within 5 days of symptom onset. Regulatory authorities recommended similar criterion for inclusion in this phase 2 study, even though the patient population was significantly different. All patients were vaccinated and not at risk for progression to severe disease. In this study, 80% of patients was randomized and initiated treatment within 3 days of symptom onset, yet we observed multiple instances of patients rapidly becoming undetectable for SARS-CoV-2 by IVA, RAT, and/or qRT-PCR within 1–2 days after randomization (i.e., 4–5 days after symptom onset). By contrast, in the phase 2 study of ensitrelvir, which used a cell culture assay with a similar lower limit of detection (0.8 TCID_50_/mL), approximately 50% of patients in the placebo group remained positive by IVA on Day 5 (15). The majority of patients in the ensitrelvir study were infected with the Omicron BA.1 variant of SARS-CoV-2, whereas the majority of patients in our study were infected with the BQ.1/BQ.1.1 Omicron variants, suggesting that these newer variants are clearing more rapidly. Given that the onset of COVID-19 symptoms often trails SARS-CoV-2 replication (20), the initiation of pomotrelvir in this study was likely to have occurred after the peak of viral infection, which would contribute to the lack of antiviral activity of pomotrelvir in regard to viral replication and symptom alleviation/resolution. Treatment initiation within 3–5 days after symptom onset is also inconsistent with current CDC guidelines, which state that patients stop isolating 5 days after symptom onset or a positive COVID-19 test, a time at which treatment would presumably no longer be necessary. In this study, approximately 30% of patients had a positive SARS-CoV-2 IVA test on Day 3 of treatment, which corresponded to 5–6 days after symptom onset. These results suggest that there is a potential for transmission after the recommended 5 days of isolation by the CDC.

3C-like protease inhibitors of SARS-CoV-2 have demonstrated mixed results in other studies of patients with mild-to-moderate COVID-19, who were not at high risk of progressing to severe disease. In an earlier study conducted in Japan, patients treated with ensitrelvir for 5 days demonstrated a significant decrease in viral titer and viral load of the Delta variant of SARS-CoV-2 compared with placebo on Day 4, with a trend toward improvement of some respiratory symptoms (21). Conversely, in a separate US study run concurrently with our study, patients treated with EDP-235 for 5 days also demonstrated no differences in SARS-CoV-2 viral RNA decline or infectious viral load, nor were there any differences in the time to improvement of 14 targeted COVID-19 symptoms compared with placebo (https://ir.enanta.com/node/12106/pdf). Notably, the ensitrelvir study was conducted at an earlier time in Japan, and the majority of patients were infected with the Delta variant of SARS-CoV-2, while this study and the EDP-235 study were conducted in the United States during the period of Omicron infection.

These results suggest that currently, infection of immunocompetent, previously vaccinated, and infected individuals not at risk for serious disease with the SARS-CoV-2 Omicron variant results in a relatively mild and rapidly cleared viral infection similar to seasonal influenza and common cold coronavirus infections. During this period of Omicron infection in a population that is vaccinated and/or previously infected, with robust immune responses, the lack of effect demonstrated in phase 2 studies of SARS-CoV-2 protease inhibitors for the treatment of COVID-19 suggests that future studies designed to demonstrate antiviral efficacy should explore the initiation of treatment within 24–36 hours after symptom onset in order to detect an antiviral effect on parameters of viral infection. Such a study design would be consistent with currently available treatments for influenza virus, which specify that treatment should be initiated within 48 hours after symptom onset. In the NDA-enabling Phase 3 study of baloxavir marboxil (Xofluza), the majority of patients were enrolled within 24 hours of symptom onset, and the difference in the time to alleviation of symptoms between the baloxavir marboxil group and the placebo group was greater when treatment was initiated within 24 hours after symptom onset (median difference, 32.8 hours; *P* < 0.001) (22).

## MATERIALS AND METHODS

### Study design

A phase 2, multicenter, randomized, double-blind, placebo-controlled study that evaluated the antiviral activity, clinical efficacy, and safety of orally administered pomotrelvir compared with placebo.

### Patients

Patients were non-hospitalized, symptomatic males and females from 18 to <65 years old with COVID-19, who were not at high risk of progressing to severe disease, with onset of COVID-19 symptoms within 5 days prior to randomization, a positive SARS-CoV-2 test within 24 hours prior to randomization, and at least two symptoms of acute SARS-CoV-2 infection at randomization. Patients had a primary COVID-19 vaccination series (and any booster) at least 3 months prior to randomization.

### Randomization and blinding

Patients were randomized 2:1 to the pomotrelvir or placebo treatment group and assigned a unique subject number via an interactive voice and web response system. Randomization was stratified based on SARS-CoV-2 positive direct test diagnosis ≤3 days versus >3–5 days from the first onset of COVID-19 symptom(s) and if patients received primary vaccination series alone versus any COVID-19 booster doses. Patients, investigators, and all internal and external personnel directly involved in the conduct of the study were blinded to treatment assignment.

### Treatment and procedures

Following randomization on Day 1, subjects initiated dosing with either pomotrelvir 700 mg (2× 350 mg tablets twice daily for a total daily dose of 1,400 mg) or placebo (two placebo tablets twice daily for a total daily dose of four tablets). All treatment was taken with food for 5 days (10 total doses). Virologic and clinical assessments were conducted at the study site on Days 1, 2, 3, 5, 10, 15, and 28, as well as rebound and early termination visits if appropriate.

### Outcomes and assessments

The primary endpoint was the proportion of patients with undetected infectious SARS-CoV-2 from MT nasal swabs on Day 3 of treatment. Secondary endpoints through Day 28 included SARS-CoV-2 infection by IVA, RAT, and qRT-PCR; symptom alleviation and resolution; COVID-19-related hospitalization or death from any cause; and COVID-19-related medical visits other than hospitalization.

### Virologic assessments

Two sets of MT nasal swab samples were collected at each study visit; one for the determination of infectious viral titer [median tissue culture infectious dose (TCID_50_)], viral load (RNA log_10_copies/mL), and whole-genome sequencing, and the other sample was used for RAT. Nasal swabs were collected using a universal viral transport collection kit with flexible minitip flocked swab from Becton, Dickinson and Company (Franklin Lakes, NJ, USA). A saliva sample was also collected for the determination of viral load using the OMNIgene ORAL Collection kit (DNA Genotek, Ontario, Canada). All sample collections were conducted by clinic personnel. RAT was conducted at the study site using the Pilot COVID-19 At-Home Test (Roche Diagnostics, Indianapolis, IN, USA). The cell-based IVA from MT nasal swab samples was conducted by Viroclinics-DDL (Rotterdam, Amsterdam, the Netherlands) using validated protocols. The qRT-PCR (MT nasal swab and saliva samples) and whole-genome sequencing assays (MT nasal swabs) were conducted by Eurofins-Viracor (Lenexa, KS, USA) using validated protocols. Details of the protocols are provided in the supplementary materials.

### Clinical assessments

#### Patient-reported outcomes

Patients completed a symptom diary every day from screening through Day 28 to grade the severity of 14 targeted COVID-19 symptoms on a four-point scale (i.e., absent, mild, moderate, and severe). Patients reported any COVID-19-related hospitalizations or acute/critical care visits.

#### Safety

Patient safety was monitored throughout the study. Adverse events were coded using the Dictionary for Regulatory Activities (v25.1), and severity was graded using the Division of AIDS Table for Grading the Severity of Adult and Pediatric Adverse Events (v2.1 July 2017).

#### Serology testing

SARS-CoV-2 N antigen-antibody status at baseline was evaluated from plasma samples using the Roche Elecsys Anti-SARS-CoV-2 assay.

#### Statistical analysis

A sample size of 210 patients accounted for the expectation that approximately 60% of randomized patients would be SARS-CoV-2 negative by IVA at baseline and, therefore, excluded from the mITTV analysis set. The sample size was sufficient to detect a greater than 19% absolute difference in the proportion of subjects below the limit of detection for SARS-CoV-2 (99% versus 81%) with at least 80% power, assuming an alpha of 0.05 and 40% of randomized subjects having detectable SARS-CoV-2 at baseline. Primary and secondary endpoints were analyzed using SAS Version 9.4 (Cary, NC, USA). The intention-to-treat analysis set included all randomized patients; the modified intention-to-treat analysis set included all randomized patients with at least two symptoms consistent with COVID-19 within 5 days prior to randomization and a positive SARS-CoV-2 test within 24 hours prior to randomization, who received at least one dose of the study drug; the modified intention-to-treat virologic analysis set included a subset of the mITT analysis set who had detectable infectious SARS-CoV-2 by IVA on Day 1. The safety analysis set included all patients who received at least one dose of the study drug. The primary endpoint analysis used the mITTV analysis set; secondary and exploratory virologic and clinical endpoint analyses used the mITT analysis set. The between-group difference in the proportion of patients below the limit of detection for SARS-CoV-2 was analyzed by a strata-adjusted risk difference and corresponding 95% CIs using the Mantel-Haenszel method. Between-group comparisons of the proportion of patients with undetectable SARS-CoV-2 by RAT and qRT-PCR from MT nasal swab samples were conducted in a manner similar to the primary analysis. The time to sustained alleviation and sustained resolution of targeted COVID-19 symptoms was estimated using the Kaplan-Meier method. Between-group differences were determined using a Wilcoxon-Gehan test, and corresponding *P*-values were calculated. Treatment-emergent AEs were those with onset from the date and time of the first dose of the study drug through the date of the last dose of the study drug plus 14 days.

## SUPPLEMENTAL MATERIAL

Figure S1 spectrum.02980-23-s0001.tif Patient disposition.

Supplemental Data spectrum.02980-23-s0002.docx Supplemental text and Tables S1 and S2.

ASM does not own the copyrights to Supplemental Material that may be linked to, or accessed through, an article. The authors have granted ASM a non-exclusive, world-wide license to publish the Supplemental Material files. Please contact the corresponding author directly for reuse.
