# Supplementary figures and images for: SARS-CoV-2 viral dynamics in a placebo-controlled phase 2 study of patients infected with the SARS-CoV-2 Omicron variant and treated with pomotrelvir
Source: Microbiol Spectr. 2024 Jan 10;12(2):e02980-23. doi: 10.1128/spectrum.02980-23 (PMC10845961; doi:10.1128/spectrum.02980-23)

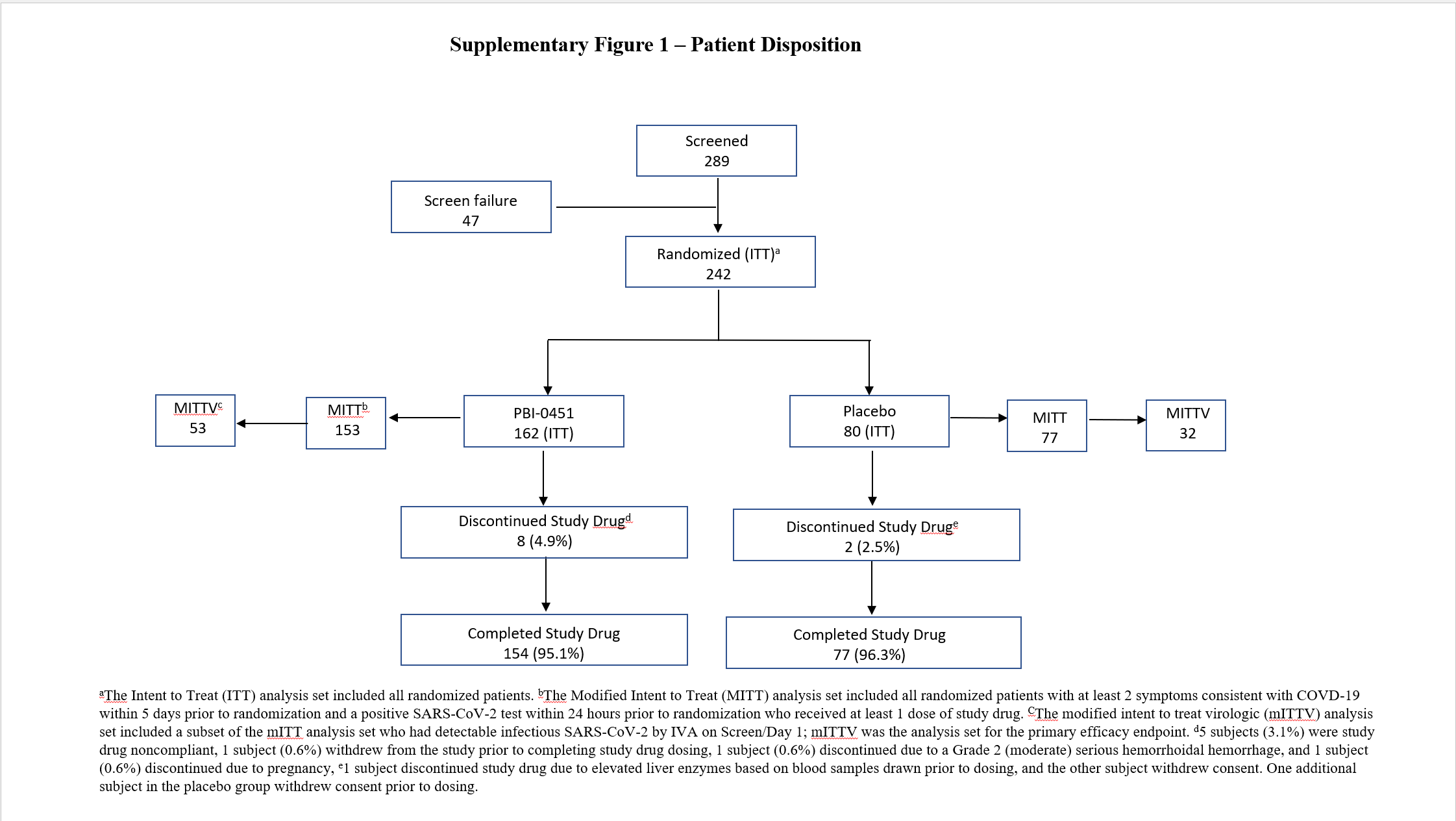

Supplement: Figure S1 — Patient disposition. [file spectrum.02980-23-s0001.tif]
