# Supplementary material for: SARS-CoV-2 viral dynamics in a placebo-controlled phase 2 study of patients infected with the SARS-CoV-2 Omicron variant and treated with pomotrelvir
Source: Microbiol Spectr. 2024 Jan 10;12(2):e02980-23. doi: 10.1128/spectrum.02980-23 (PMC10845961; doi:10.1128/spectrum.02980-23)
Supplement: Supplemental Data — Supplemental text and Tables S1 and S2. [file spectrum.02980-23-s0002.docx]

## Supplementary Data

## Virologic Assessments

1. Determination of infectious virus titer
2. A cell-based IVA from MT nasal swab samples collected on Screen/Day 1, 2, 3, 5, 10, and 15,
3. and any rebound or early termination visit. In this assay, 8- to 10-fold dilutions of the samples
4. were plated on Vero-E6/TMPRSS2 cells, and infectivity was evaluated after 6 days by
5. nucleoprotein immunostaining. The assay was originally validated using the SARS-CoV-2
6. Wuhan strain, and validation has been extended to include other SARS-CoV-2 variants of
7. concern (e.g., Alpha, Beta, Epsilon, Mu, Gamma, and Omicron variants) with similar results.
8. Validation parameters included accuracy, precision, reproducibility, linearity, lower limit of
9. quantitation, upper limit of quantitation, and stability. The validated range of the assay for
10. samples collected from MT nasal swabs is 1.0-5.4 log_10_ TCID_50_/mL, and the limit of detection is
11. 0.75 log_10_ TCID_50_/mL; sample titers less than the limit of detection of the assay were transformed
12. into 0.375 log_10_ TCID_50_/mL for statistical purposes.
13. Determination of SARS-CoV-2 RNA by qRT-PCR
14. A qRT-PCR assay was conducted on MT nasal swab and saliva samples collected on Screen/Day
15. 1, 2, 3, 5, 10, 15, 28, and any rebound or early termination visit. The primers used in the assay
16. target 2 regions of the SARS-CoV-2 N gene were designed and developed by Eurofins
17. Viracor. The primers and probes were designed using the SARS-CoV-2 Wuhan strain
18. (NC_045512.2). Validation parameters included analytical specificity, linearity and dynamic
19. range, lower limit of detection, lower limit of quantitation, intraassay and interassay precision
20. (reproducibility), and analyte stability. The validated range of the assay for samples collected
21. from MT nasal swabs is 714-7.1 × 10^7^ copies/mL and the limit of detection is 299 copies; sample
22. viral loads less than the limit of detection were transformed into 1.238 log_10_ copies/mL/mL for
23. statistical purposes. The validated range of the assay for samples collected from saliva is 714–7.1
24. ×10^7^ copies/mL, and the limit of detection is 149 copies/mL, sample viral loads less than the
25. limit of detection were transformed into 2.173 log_10_ copies/mL for statistical purposes.
26. SARS-CoV-2 sequencing
27. Whole genome sequencing was conducted on all available MT nasal swab samples with
28. sufficient levels of viral RNA (i.e., > 3.85 log_10_ copies/mL). The 30 kb viral genome is amplified
29. in approximately 400 base pair amplicons that are then used as input for next-generation
30. sequencing library preparation for short-read sequencing. The sequencing assay was conducted
31. based on the ARTIC protocols using the Illumina platform (https://artic.network). The assay was
32. validated using the Wuhan strain (NC_045512.2), it has not been fully validated against all
33. SARS-CoV-2 variants of concern. Validation parameters included the limit of detection,
34. intraassay and interassay precision, and accuracy. The default variable allele frequency
35. established for the assay is 50%; the minimum coverage for variants of 6.7% of the sample viral
36. population was used for exploratory endpoints.
37. **Supplementary Table 1 Baseline demographics and clinical characteristics (ITT analysis set)**

| **Variable** | **Pomotrelvir (n = 162)** | **Placebo (n = 80)** | **Total (N = 242)** |
| --- | --- | --- | --- |
| Age, mean (SD), years | 42.7 (12.3) | 42.4 (11.8) |  |
| Male, n (%) | 77 (47.5) | 38 (47.5) | 115 (47.5) |
| Race, n (%) |  |  |  |
| White | 134 (82.7) | 71 (88.8) | 205 (84.7) |
| Black or African American | 21 (13.0) | 8 (10.0) | 29 (12.0) |
| Other (Asian, American Indian or Alaska, Native, Native Hawaiian or Other Pacific Islander | 7 (1.5) | 1 (1.3) | 8 (3.3) |
| Ethnicity |  |  |  |
| Hispanic or Latino | 129 (79.6) | 64 (80.0) | 193 (79.8) |
| Body mass index (SD), kg/m2 | 26.2 (2.9) | 27.5 (4.1) |  |
| COVID-19 vaccination booster | 64 (39.5) | 33 (41.2)) | 97 (40.1) |
| SARS-CoV-2 N antigen antibody, n (%) |  |  |  |
| Positive | 111 (68.5) | 56 (70.0) | 167 (69.0) |
| Time from symptom onset to randomization, n (%) |  |  |  |
| ≤ 3 days | 134 (82.7) | 64 (80.0) | 198 (81.8) |
| > 3 days to ≤ 5 days | 28 (17.3) | 16 (20.0) | 44 (18.2) |
| Targeted COVID-19 symptoms |  |  |  |
| Cough | 148 (91.4) | 67 (83.8) | 205 (84.7) |
| Stuffy or runny nose | 145 (89.5) | 67 (83.8) | 212 (87.6) |
| Low energy or tiredness (fatigue) | 140 (86.4) | 64 (80.0) | 204 (84.3) |
| Headache | 131 (80.9) | 57 (71.3) | 188 (77.7) |
| Sore throat | 121 (74.7) | 63 (78.8) | 184 (76.0) |
| Muscle or body aches | 122 (75.3) | 60 (75.0) | 182 (75.2) |
| Feeling hot or feverish | 98 (60.5) | 47 (58.8) | 145 (59.9) |
| Chills or shivering | 98 (60.5) | 42 (52.5) | 140 (57.9) |
| Shortness of breath (difficulty breathing) | 68 (42.0) | 34 (42.5) | 102 (42.1) |
| Nausea | 51 (31.5) | 31 (38.8) | 82 (33.9) |
| Sense of taste | 47 (29.0) | 23 (28.8) | 70 (28.9) |
| Sense of smell | 43 (26.5) | 24 (30.0) | 67 (27.7) |
| Diarrhea | 35 (21.6) | 16 (20.0) | 51 (21.1) |
| Vomit (throw up) | 10 (6.2) | 6 (7.5) | 16 (6.6) |
| Positive SARS-CoV-2 by IVA, n (%) | 53 (32.7) | 32 (40) | 85 (35.10 |
| Viral titer (log10 TCID50/mL), mean (SD) | 2.01 (0.983) | 2.13 (1.548) |  |
| Nasal swab Viral RNA (log10 copies/mL), mean (SD) | 5.34 (2.148) | 5.13 (2.465) |  |
| Saliva Viral RNA (log10 copies/mL), mean (SD) | 4.71 (1.643) | 4.93 (1.401) |  |

1. **Supplementary Table 2. Median time to sustained alleviation of targeted COVID-19 symptoms**

| **Targeted COVID-19 Symptoms** | **Sustained Symptom Alleviation** | | **Sustained Symptom Resolution** | |
| --- | --- | --- | --- | --- |
|  | **Pomotrelvir (n = 153)** | **Placebo (n = 77)** | **Pomotrelvir (n = 153)** | **Placebo (n = 77)** |
| **14 Symptoms** | | | | |
| Events^a^, n (%) | 143 (93.5) | 69 (89.6) | 132 (86.3) | 66 (85.7) |
| Censored^b^, n (%) | 10 (6.5) | 8 (10.4) | 21 (13.7) | 11 (14.3) |
| Median Days to Alleviation^c^ (95% CI) | 8.0 (7.00,  9.00) | 8.0 (6.00,  9.00) | 10.0 (8.00,  11.00) | 11.0 (8.00,  11.00) |
|  | **Pomotrelvir**  **(n = 153)** | **Placebo**  **(n = 77)** | **Pomotrelvir**  **(n = 153)** | **Placebo**  **(n = 77)** |
| p-value^d^ | 0.8625 | | 0.9252 | |
| **12 Symptoms** | | | | |
| Events^a^, n (%) | 146 (95.4) | 71 (92.2) | 132 (86.9) | 67 (87.0) |
| Censored^b^, n (%) | 7 (4.6) | 6 (7.8) | 20 (13.1) | 10 (13.0) |
| Median Days to Alleviation^c^ (95% CI) | 7.0 (6.00,  8.00) | 7.0 (6.00,  8.00) | 7.0 (6.00, 8.00) | 9.0 (7.00,  11.00) |
| p-value^d^ | 0.6676 | | 0.9574 | |
| **5 Symptoms** | | | | |
| Events^a^, n (%) | 147 (96.1) | 71 (92.2) | 133 (86.9) | 67 (87.0) |
| Censored^b^, n (%) | 6 (3.9) | 6 (7.8) | 20 (13.1) | 10 (13.0) |
| Median Days to Alleviation^c^ (95% CI) | 6.0 (6.00,  7.00) | 6.0 (6.00,  8.00) | 9.0 (8.00, 11.00) | 9.0 (7.00,  11.00) |
| p-value^d^ | 0.2046 | | 0.9514 | |
| **Respiratory Symptoms** | | | | |
| Events^a^, n (%) | 147 (96.1) | 71 (92.2) |  |  |
| Censored^b^, n (%) | 6 (3.9) | 6 (7.8) |  |  |
| Median Days to Alleviation^c^ (95% CI) | 6.0 (5.00,  7.00) | 8.0 (6.00,  8.00) |  |  |
| p-value^d^ | 0.4129 | | 0.6843 | |

1. CI=confidence interval
2. Sustained alleviation of symptoms was the time that all targeted symptoms were concurrently
3. alleviated for 2 consecutive days from their status at baseline (i.e., when all symptoms scored as
4. moderate or severe at baseline were scored as mild or absent, and all symptoms scored as mild or
5. absent at baseline were scored as absent for 2 consecutive days).
6. a Patients who met sustained alleviation of symptoms through Day 28.
7. b Patients who did not meet sustained alleviation of symptoms through Day 28 were
8. censored as follows:
9. • Patients who discontinued from the study prior to or on Day 28 were censored at their
10. study discontinuation date.
11. • Patients who had monotonic missing data (i.e., a sequence of missing values through
12. Day 28) were censored at the date of the last symptom diary completion.
13. • Patients who completed their symptoms diary through Day 28 but did not meet
14. alleviation criteria were censored at Day 29.
15. c Kaplan-Meier estimate
16. d Wilcoxon-Gehan test
